# The Effects of Transformational Leadership, Organizational Innovation, Work Stressors, and Creativity on Employee Performance in SMEs

**DOI:** 10.3389/fpsyg.2022.772104

**Published:** 2022-04-21

**Authors:** Jawaria Nasir, Rashidah M. Ibrahim, Muhammad Arslan Sarwar, Binesh Sarwar, Waleed Mugahed Al-Rahmi, Fahad Alturise, Ahmad Samed Al-Adwan, Mueen Uddin

**Affiliations:** ^1^Faculty of Business & Management, Universiti Sultan Zainal Abidin, Kuala Nerus, Malaysia; ^2^Faculty of Management and Administrative Sciences, University of Gujrat, Gujrat, Pakistan; ^3^Department of Management Sciences, COMSATS University Islamabad, Sahiwal, Pakistan; ^4^Faculty of Social Sciences & Humanities, School of Education, Universiti Teknologi Malaysia, Johor Bahru, Malaysia; ^5^Department of Computer, College of Science and Arts in ArRass Qassim University, Ar Rass, Saudi Arabia; ^6^Electronic Business and Commerce Department, Business School, Al-Ahliyya Amman University, Amman, Jordan; ^7^School of Digital Science, Universiti Brunei Darussalam, Gadong, Brunei Darussalam

**Keywords:** work stressor, transformational leadership, creativity, organizational innovation, employee performance

## Abstract

**Purpose of the Study:**

The significance of creativity and performance in the workplace has been illustrated on various occasions. This study aims to find out if there is a link between transformative leadership, organizational innovation, psychological issues such as hindrance and challenge stressors, and employee creativity and employee performance. There is still a lack of awareness of the factors that influence employee performance in small and medium businesses (SMEs) in Pakistan. Pakistan’s SMEs have struggled to survive in their early years, with an initial failure rate of 90 percent to 95 percent.

**Methodology:**

The relationship between hindrance and challenge stressors, organizational innovation, transformational leadership, employee creativity, and their effect on overall employee performance is established through employing Structural Equation Modeling (SEM). In this study, constructs were developed from existing theories, hypotheses were generated, data were collected from 424 SME employees, and SEM analysis was conducted to prove the suggested hypothesis. The employees of SMEs are the research study’s unit of analysis.

**Findings:**

The findings of this study demonstrated that challenge stressors, transformational leadership, and employee creativity all had positive and significant effects on employee performance.

**Originality/Value:**

This is one of the first studies to study and extends existing understanding of psychological research in this manner and following correlations in a developing country, Pakistan: the links between transformational leadership and employees’ perception of creativity and performance along with organizational innovation and work stressors. Based on theoretical considerations, a model is proposed, and hypotheses are established and explored. The findings of this study can help businesses increase employee performance by informing employee performance improvement methods. Business executives might learn more about how to engage and motivate employees to improve their performance.

## Introduction

The primary purpose of human resources in an organization is to successfully manage its employees by fostering good attitudes such as enhanced productivity, work satisfaction, enthusiasm, and organizational citizenship behavior, and minimizing negative employee attitudes such as increased turnover, tardiness, and disruptive workplace behavior. These criteria jointly describe an employee’s performance at work. Employee performance is linked to an organization’s overall performance and success (Al Khajeh, 2018). Organizations must therefore ensure that their staff is driven to perform at their maximum level of performance. It is no secret that leadership has been increasingly popular in recent years as a way to effectively manage people and the business at large. This concept of personnel administration is slowly giving way to the concept of human resource management (HRM). Integrating modern leadership styles into successful management of people and enhancing employee performance should be the top priority for any company. When it comes to employees, several leadership styles are used depending on how much guidance, empowerment, and decision-making power they are given (Mohiuddin, 2017). Along with effective leadership, continuous innovation is the basic driving force for managing and motivating the employees and organizational performance.

The study focuses on the importance of transformational leadership, organizational innovation, work stressors, and creativity on employee performance in the manufacturing sector of Pakistan, as well as the performance of small and medium companies (SMEs). To address performance issues in SMEs, Pakistan has implemented a “one-size-fits-all” policy. This, however, has made no difference to the status of development of SMEs, as the majority of these businesses have failed to survive past their first year of operation. In this context, the focus of this research was on the impact of leadership, innovation, and stressors on the performance of employees of SMEs in Pakistan’s manufacturing industry. Pakistan has focused on SMEs, particularly the manufacturing sector, since 2004 in order to achieve long-term economic growth. In Pakistan, however, 95 percent of SMEs fail within the first year of operation (Nasir et al., 2020a). Similarly, SMEs’ productivity in large industries, such as textiles, is on the decline. Furthermore, SMEs have a poor level of innovation and technological advancement. Experts also believe that a lack of innovation, leadership, and performance competencies is a major cause of these issues (Nasir et al., 2020a). Despite the crucial role of employees in SMEs, there is a scarcity of in-depth study on this topic in Pakistan.

Innovation has long been recognized as one of the most important aspects of a company’s success and a country’s economic progress. As a result, academics have traditionally placed a greater emphasis on comprehending the elements that enable or inhibit innovation in organizational contexts (Shafique et al., 2019). Successful innovation, on the other hand, is dependent on a number of individual and organizational elements. For example, a number of academics have identified creativity—the generation of useful and novel ideas—as a prerequisite for innovation—the successful implementation of creative ideas—across the organization. Many more studies have reported the significance of different leadership styles in achieving enhanced innovation and employee performance. Despite the fact that several studies have been conducted on the relationship between different leadership styles, creativity, organizational innovation, and employee performance, this field of study remains underdeveloped (Al Harbi et al., 2019; Yu et al., 2019).

In today’s fast-paced and highly competitive economic landscape, businesses must invest in creativity and innovation to remain competitive and sustainable. Employee perceptions of leadership, procedures, and policies that support or impede creativity and innovation in the organization must be prioritized as enablers of inventive outputs. To get a competitive advantage, companies need to be able to innovate and be creative. And it can contribute to enhance employee performance and reduce stressor (He et al., 2019). Scholars from a variety of fields have attempted to understand the essential aspects that influence creativity and invention. For example, Mumford et al. (2002) identify a wide range of elements such as climate, individual performance capacities, strategy, and structure in their review. Prior research on antecedents of creativity and innovation has focused on personal (leadership capabilities) and contextual (supportive atmosphere for innovation) aspects (Wang et al., 2014). The authors Xian et al. (2020) believe that further investigation is needed to better understand the elements that influence employees’ creative and organizational innovative behavior for enhancing overall employee performance. As a result, both leadership and organizational innovation are examined in this research, along with hindrance and challenge stressors (Nasir et al., 2020a).

Transformational leadership was chosen from among all available leadership theories because it has been shown to generate and enhance creativity and innovation. In this context, Bass (1985) described a transformational leader as someone who motivates subordinates to go above and beyond their expectations. A transformational leader is dynamic, proactive, and capable of influencing themselves and their followers to embrace change (Nasir et al., 2020a). According to Ergeneli et al. (2007), transformational leaders encourage their employees to go beyond their own self-interests for the good of their organizations. Despite the theoretical explanation, there is little evidence supporting the correlations mentioned earlier (Xian et al., 2020).

As defined by researchers, organizational innovation is how it feels to be a member of a corporation. It reflects the behaviors and reactions of employees to what the workplace assumes and values (Gundry et al., 2015). The organizational innovation construct reflects employees’ shared values to the behaviors they believe are being predicted, encouraged, and recognized. A business environment for creative and innovative behavior reflects employees’ impressions of organizational practices, procedures, and policies, as well as ways of interacting with one another that foster or inhibit creative and innovative behavior (Shafique et al., 2019).

Workers in the organization are generally stressed when they have to strengthen ties with coworkers and supervisors, possibly due to work overload, excess activities formed due to inter-role disputes, unattainable deadlines, a lack of promotional incentives, role ambiguity and creativity, and long working hours. Even senior management’s rotation of staff causes tension in the workplace. Workplace stress is considered to be caused mostly by globalization, technical innovation, and unhealthy competitiveness on a global scale (Miao and Cao, 2019). Stress, throughout particularly, is a major issue that produces a tense and inflexible work atmosphere for employees that causes not only tension, lack of confidence, and physical disorders but also diminishes their commitment, sense of accomplishment, motivation, and work performance (Barello et al., 2020; Li et al., 2020).

Researchers observed that job stress is linked to negative outcomes such as dishonesty, low morality, weariness, absences, and job—or voluntary turnover (Le, 2020), which is detrimental to organizations and their members. Despite this, the literature on the association between work stress and employment outcomes is largely conflicting and contradictory. The link between the two factors has been found to be very insufficient in other investigations (Chauhan et al., 2019). In summary, the purpose of this study is to provide empirical answers to the following research questions: Are there significant relationships between transformational leadership, organizational innovation, and work stressors with employees’ creativity and employee performance in the context of small, medium enterprises.

Pakistan has a pressing need to increase the value added to its products, given the current state of the country’s SMEs. 1 million bales of cotton sold in Pakistan for USD 1 billion, but India sold 1 million bales for USD 2 billion, and China sold 1 million bales for USD 4 billion. Despite Pakistan’s economy’s reliance on small- and medium-sized enterprises (SMEs), the sector has a number of flaws. The vast majority of SME businesses are small- and medium-sized. Most small businesses are run by their owners. These businesses are unlikely to expand or create many new jobs in the near future. The primary goal of these companies is still survival, not expansion. The most pressing issue for fast-growing companies is attracting and retaining talented employees. SMEs are unable to attract highly skilled workers due to a lack of money.

## Literature Review and Development of Hypotheses

### Organizational Innovation and Employees’ Creativity and Employee Performance

Technology and administrative innovations cannot be achieved without innovation, which is the missing link between HRM practices and creativity as an end-product (Chaubey and Sahoo, 2019). Innovation practices give crucial inputs for the later creation and implementation of creative products and services and new work processes and procedures in a business (Dedahanov et al., 2017). According to Shalley and Gilson (2004), individuals are the primary source of innovation. Initiating information for organizational innovation comes from individual employees who develop innovative ideas (Tu and Lu, 2016). Employees who are creative are more likely to come up with new product ideas as well as innovative ways to use existing products, processes, and methods (Rasheed et al., 2017). As a result, such employees might be regarded as an organization’s ultimate source of high creative performance. Furthermore, these personnel not only come up with innovative ideas, but also plan how to put them into action. Furthermore, in addition to becoming idea champions, creative people are more likely to act as role models and inspire other employees at work, transforming them into idea generators. The innovative ideas of creative persons can also be communicated to other employees in the organization for self-improvement and application, which can lead to the development and advancement of organizational innovation and overall employee performance (Shalley and Gilson, 2004; Abbas et al., 2020). Individual creativity is thus expected to contribute to innovative outcomes at the organizational level through the generation and implementation of new ideas. According to Hsu and Chen (2017), there is a link between new process innovation and employee performance. Higher productivity has a strong positive link with innovation and the first entry. The role of innovation in European countries was investigated by Ghisetti et al. (2015) and Abbas et al. (2020), and the economic results revealed that innovation had a considerable positive impact on performance. According to Mahmood et al. (2019), process innovation leads to increased productivity and greater organizational performance. Moreover, Hall et al. (2009) revealed that both product and process innovation have beneficial impacts on employee performance; also, process innovation has a stronger influence on employee productivity. As a result, it is assumed that as:

*Hypothesis* 1: Organizational Innovation is positively related to employees’ creativity.

*Hypothesis* 2: Organizational Innovation is positively related to employees’ performance.

### Transformational Leadership and Employees’ Creativity and Employee Performance

It is widely accepted that transformational leadership is a prominent concept in management literature due to its collaborative and inspirational style of leadership. Subordinates of transformational leaders work longer hours and produce more than is expected of them (Bass, 1985). When they require help, they guide them, refine their skills, impart knowledge to them, and treat every one of them equally (Miao and Cao, 2019). By definition, “it is a kind of leadership in which organizations are managed around a purpose in ways that inspire and advance the aspirations of employees” (Burns et al., 1978, p. 32). As a result, transformational leaders strive to develop their followers’ knowledge and abilities and raise their aspirations and requirements. As a result, the subordinates become more united and change their goals and values. According to previous study on this topic, companies’ success is correlated with employee performance (Rubera and Kirca, 2012). Researchers began incorporating the concept of innovation with creativity in the late 1990s. Businesses were compelled by the relationship between these two ideas to recognize the need to develop employees’ performance (Al Harbi et al., 2019).

According to Mumford et al. (2002, p. 705) report, “creativity and innovation, the production of new ideas and their application, are now viewed as key goals of many organizations, and as having a major influence on employee performance.” To be creative, you need to have a unique perspective on things. Individuals are the primary source of innovation in a company (Shalley and Gilson, 2004). It is claimed that the employees’ inventiveness gives the motivation for innovation. Employees that are creative tend to discover potential for new goods or new ways to use existing methods, coming up with new ideas to solve work-related difficulties, and establishing sufficient strategies for implementing these new ideas and plans. Creative employees, according to Mahmood et al. (2019), come up with new and helpful ideas for products, methods, and practices. Leaders with a transformative leadership style are recognized as the key drivers of staff creativity and innovation. Transformational leaders, on the other hand, encourage their subordinates to think creatively, analyze their challenges from multiple perspectives, and come up with new and innovative solutions. Transformational leaders have a high level of confidence and trust among their employees, according to Miao and Cao (2019). Since employees will be motivated and supported to take chances in order to complete their tasks, this trust will also inspire critical thinking and stimulate them to take risks most of the time at work. Transformational leaders encourage their subordinates to take chances, and they take responsibility for the outcomes of their subordinates’ actions. When employees receive this support, their mindset changes and that encourages them to participate in creative and innovative work processes (Suifan et al., 2018). Because they are willing to take on more challenges, transformational leaders boost the creative and innovative skills of their staff (Ribeiro et al., 2018; Mahmood et al., 2019). According to Yunus and Anuar (2012), transformative leaders challenge those they lead and inspire them to look for new and innovative approaches to their performance.

So it brings to our hypothesis as:

*Hypothesis* 3: Transformational leadership is positively related to employees’ creativity.

*Hypothesis* 4: Transformational leadership is positively related to employees’ performance.

### Hindrance Work Stressor and Employees’ Creativity and Employee Performance

Stress from a stressor-strain perspective usually associates negatively with the results of employee work. This view advocates that stressors may lead to stresses (e.g., fatigue and exhaustion) caused by emotional and cognitive effort in the evaluation and trying to cope processes and thus reduce the energy used to carry out tasks (Antwi et al., 2019). The “hindrance stressors” component included organizational politics, red tape, role uncertainty, and fears about job security. It also featured severe demands that managers saw as unnecessary impediments to human development and goal achievement (Nasir et al., 2020b). Importantly, the findings of regression analysis revealed that hindrance stresses were inversely connected with job satisfaction, creativity, and overall performance (LePine et al., 2005). This form has been defined as a hindrance stressor because stressful demands are seen by people as obstacles to personal growth and achievement of objectives. Hindrance stressors are considered to manipulate and threaten as opposed to challenge stressors. Previous research showed that hindrance stressors are negatively linked to organization’s results (e.g., worker satisfaction, organizational engagement, and performance; LePine et al., 2005; Podsakoff et al., 2007). When it comes to inventive behavior, hindrance stressors may affect idea development and implementation differently. Cowen discovered a link between psychological threat and rigid thinking. Furthermore, increased unpredictability was observed to reduce creative performance (Byron et al., 2010). As a result, it is possible that barrier pressures obstruct the creation of creative thoughts. Idea implementation, as opposed to idea generation, refers to putting creative ideas into action (Song et al., 2017). As they challenge and violate the existing frameworks of practices and the status quo in the organization, creative ideas are likely to be met with skepticism and criticism (Janssen and Van Yperen, 2004; Barello et al., 2020).

To summarize, we hypothesize as:

*Hypothesis* 5: Hindrance work stressor is negatively related to employees’ creativity.

*Hypothesis* 6: Hindrance work stressor is negatively related to employees’ performance.

### Challenge Work Stressor and Employees’ Creativity and Employee Performance

The next hypothesis of the study is challenge-related stress is positively related to employee performance, and challenge-related stress is also positively related to employee creativity. As per literature challenge-oriented stress helps employees to boost their performance and also provides intrinsic motivation (Song et al., 2017). Bakker and van Woerkom (2017) state that stress arises when someone perceives that the parameters for an outside situation go beyond their expected capacity to cope. According to the stressor-strain viewpoint, work stressors are the factors that trigger the stress process, and types of strain, such as tension, anxiety, and fatigue, are the proximal outcomes of this process (Karatepe et al., 2018). Recent research has shown that while all stressors are stress-causing, but also different forms of stressors have different affective and behavioral responses. Challenge stress generates optimistic emotions and an active coping style (Crawford et al., 2010; Li et al., 2020). Positive impacts on organizational outcomes such as employee performance, employee creativity, and efficiency were noticed. Innovative actions can enable workers to enhance fitness with high job challenges by creating, supporting, and developing ideas for changing themselves or the workplace (Maslach and Leiter, 2017). These results indicated that the development and implementation of ideas could function as useful counter-challenge stress strategies. It concludes our next hypotheses that as:

*Hypothesis* 7: Challenge work stressor is positively related to employees’ creativity.

*Hypothesis* 8: Challenge work stressor is positively related to employees’ performance.

### Employees’ Creativity and Employee Performance

The learning orientation promotes both adaptive and generative learning, while creativity favors generative learning. This means that learning orientation has a bigger impact. To paraphrase, an organization’s learning orientation and employee innovative thinking and performance are encouraged by a creative climate (Naderi et al., 2019). In accordance with Barrett et al. (2005), the findings suggest that a creative climate has a favorable impact on employee performance. Redmond et al. argue that the person is the primary basis of any innovative idea, creating a firm basis for corporate innovation (Shalley and Gilson, 2004). Personnel creativity is therefore a source of raw material for innovation (Ximenes et al., 2019). Employees who are creative are more likely to spot new product potential. They may discover new applications for current procedures or equipment, or they may come up with fresh but workable work-related concepts (Zhou et al., 2018). These individuals are more likely to come up with innovative solutions to issues and champion new ideas to others, and establish suitable plans for putting new ideas into action (Miao and Cao, 2019). Creative employees come up with new and helpful ideas for products, processes, and procedures. Furthermore, by serving as role models for the rest of the business, these individuals may have a spillover effect (Zeb et al., 2019). According to Shalley and Gilson (2004), new ideas generated by creative employees can be transferred to other employees in the business for use and development. As a result, through idea generation and implementation, such creativity at the personal level is expected to lead to the development of creative goods at the organizational level. Furthermore, creativity improves job-related performance of employees. Individuals become more adaptive and open to new experiences as a result of creativity, which increases novelty, usefulness independence, confidence, and willingness to take chances (Duarte et al., 2021). Employee creativity has a favorable impact on employee performance and innovation (Nasir et al., 2020a).

Hence, it generates the following hypothesis:

*Hypothesis* 9: Employee creativity effects positively to employee performance.

## Materials and Methods

### Sample and Procedures

The data for this research were collected using simple random sampling, and SEM was employed. SEM requires large sample sizes and models of more parameters to require more estimates, so bigger samples are needed for more accurate results. Awang et al. (2016) and Sarwar et al. (2020) further added that, according to a rule of thumb, the minimum sample size is 200 for studies using SEM analysis. For this research study, the target population is the SMEs of Pakistan. Small and Medium Enterprise Development Authority, SMEDA (2011) reports that the total numbers of SMEs in Pakistan are 5.2 illion. The manufacturing sector of the SMEs is focused in the current study, which is around 1 million. But only 20, 550 are registered SMEs. The information of registered SMEs was taken from the listings of the Chamber of Commerce, Directory of Industrial Establishments, Jamal Yellow pages. Also, for this study, SMEs ranging from “1 to 250” employees were selected. The targeted inquiry units were the “CEO, Managing Director, General Managers, Owner, Managers, Assistant Manager, Technicians, and Senior Staff.”

As mentioned above, 450 respondents are sampled based on the guidelines defined by Hair et al. (2014). However, to prevent a shortage of the necessary sample size and account for the incomplete and missing pattern, the framework for the necessary sample size 450 has increased by 20%. So a total of 550 questionnaires were distributed among employees of selected firms. The respondents, however, returned only 460 questionnaires, and from them, 424 useable respondents were used for further data analysis.

Out of 424 respondents, 263 were male, and almost 161 were female. It shows that Pakistani SME sector is dominant with the male population, and it also shows the overall culture of Pakistan that mostly males are doing jobs and most women are. The major portion of the respondents (48.6%) were between the ages of 30 to 40. Most of the respondents (38.2%) were having experience between 3 and 5 years. It was a mixture of the staff and workers, with 61 percent staff members and 39 percent workers. The average working hours of the respondents were between 8 and 12 h per day. Seventy-one percent of the respondents’ monthly income was between 20,000 and 50,000 PKR which is reasonable as per the Pakistani labor market but not quite enough as per first world countries.

### Measures

The final questionnaire consists of 37 items except for the demographic profile questions of respondents who were actually surveyed. The construct “Organizational innovation” consists of six questions adapted from the study of (Friedman, 2003; Hsiao and Chang, 2011). The next variable, “Transformational Leadership,” consists of seven questions adapted from the work of (Carless et al., 2000). Then, “Challenge and hindrance stressors” were measured with Cavanaugh et al. (2000) 11-item scale, six challenge stressor items, and five hindrance stressor items. “Creativity” was measured with nine items from the work of (Ettlie and O'Keefe, 1982; Cohen-Meitar et al., 2009). Finally, “Employee performance” with five items adapted from the study of (Ellinger et al., 2003; Janssen and Van Yperen, 2004).

## Results

These are the results of the study started with demographic Table 1.

**Table 1:** Demographic analysis.

Items	Options	Frequency *N* = 424	Percentage
Gender	Male	263	62
	Female	161	38
Age (Years)	20 to 29	120	28.3
	30 to 39	206	48.6
	40 to 49	35	8.3
	Over 40	63	14.9
Work experience	Less than 1 year	15	3.5
	1–3 years	132	31.1
	3–5 years	162	38.2
	More than 5 years	115	27.1
Qualification	Diploma	70	16.5
	Intermediate	129	30.4
	Graduate	141	33.3
	Post Graduate	80	18.9
	PhD/Higher Skilled Degree	4	0.9
Current working position	Managerial level	258	60.8
	Supervisor level	166	39.2
Total work hours per day	8 h or less	51	12
	8–10 h	167	39.4
	10–12 h	190	44.8
	12 h and more	16	3.8
Monthly income (PKR)	Below 20,000	170	40.1
	20,001–50,000	132	31.1
	50,001–70,000	73	17.2
	Above 70,001	49	11.6

### Measurement Model

All measurement scales have high reliability (varying from 0.87 to 0.95), indicating that none of the scale items should be eliminated. Confirmatory factor analysis was also used to look at the measures’ reliability. The AMOS-SEM approaches were used to calculate composite reliability (CR) and average variance extracted (AVE) from model estimates, as reported by Sarwar et al. (2020) and Habib and Sarwar (2021). As shown in Table 2, the measurements used in this research were within acceptable limits, model fitness was also achieved and implying that the constructs achieved reliability. Discriminant validity is a tool to improve data validity, and it is also achieved.

**Table 2:** CFA findings.

Factors	Estimate (Reflective measure)	No of items	CR (above 0.6)	AVE (above 0.5)
Organizational innovation (OINO)	0.808	7	0.921	0.624
	0.812			
	0.821			
	0.852			
	0.779			
	0.732			
	0.717			
Hindrance Stressor (WSTR)	0.833	5	0.912	0.676
	0.777			
	0.851			
	0.891			
	0.750			
Challenge Stressor (CSTR)	0.803	6	0.913	0.635
	0.821			
	0.804			
	0.770			
	0.790			
	0.793			
Transformational Leadership (TRAL)	0.778	5	0.872	0.580
	0.824			
	0.716			
	0.842			
	0.627			
Creativity (CRTV)	0.660	9	0.923	0.571
	0.690			
	0.804			
	0.782			
	0.741			
	0.807			
	0.734			
	0.760			
	0.808			
Employee Performance (EPPR)	0.820	5	0.928	0.722
	0.871			
	0.860			
	0.862			
	0.834			

CMIN/df = 2.409, p ≤ 0.00; df = 267, GFI = 0.93, AGFI = 0.91, NFI = 0.91, CFI = 0.94, RMSEA = 0.058, RMR = 0.091.

The bolded diagonal values of each construct represent a square root of the AVE and the correlation of the corresponding construct pairs are other values. The respective construct’s discriminant validity is achieved if the square root of its AVE exceeds its correlation value to other constructs in the model. In other words, discriminant validity can be accomplished if the bold diagonal values on the row and column are higher than any other value (Sarwar et al., 2020). The values in Table 3 comply with the discriminant validity criterion. Therefore, the analysis states that it maintains the discriminant validity for all constructs.

**Table 3:** Discriminant validity and correlation index summary.

	CR	AVE	MSV	MaxR(H)	CRTV	TRAL	EMPR	HSTR	OINO	CSTR
CRTV	0.923	0.571	0.401	0.926	**(0.756)**					
TRAL	0.872	0.580	0.401	0.886	0.633	**(0.761)**				
EMPR	0.928	0.722	0.375	0.930	0.612	0.322	**(0.850)**			
HSTR	0.912	0.676	0.338	0.920	0.219	0.543	0.581	**(0.822)**		
OINO	0.921	0.624	0.035	0.925	0.021	0.024	0.186	0.040	**(0.790)**	
CSTR	0.913	0.635	0.321	0.913	0.567	0.477	0.246	0.064	0.033	**(0.797)**

Values in parentheses “()” are the square root value of AVE of given variables. AVE, Average variance extracted. MSV, Maximum shared variance.

### Hypothesis Testing

**Figure 1:**
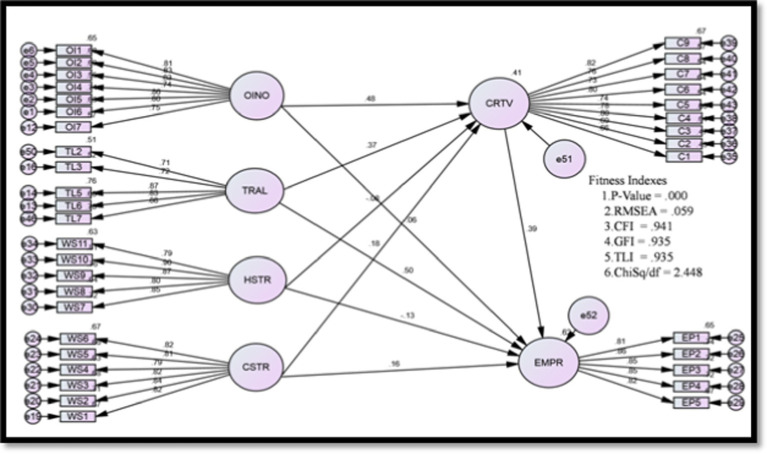
Structural Model.

Further structural model in SEM was used to test the significance of the theoretical relationships in Figure 1. The results are depicted in Table 2. All the model fit indexes are achieved and shown in Table 2. The first hypothesis proposed was organizational innovation positively affects employee performance, and according to data analysis, the beta value is (*β* = 0.06, *p* > 0.01), which is greater than one; therefore, H1 is not supported. Moreover, the next hypothesis that organizational innovation is positively related to employee creativity and outcome is (*β* = 0.48, *p* < 0.01); hence, H2 is supported. The following hypothesis, H3, which is that transformational leadership leads significantly to employee performance, is also accepted (*β* = 0.50, *p* < 0.01). The following hypothesis was transformational leadership is positively related to employee creativity, and according to results (*β* = 0.37, *p* < 0.01), H4 is also supported. Furthermore, it was proposed that Hindrance stress is negatively related to employee performance and results are (*β* = −0.13, *p* < 0.01), so H5 is also accepted. Then next, hypothesis 6 proposed was hindrance-related stress is negatively related to employee creativity, and according to results (*β* = −0.08, *p* < 0.01), H6 also supported. Additionally, next hypothesis 7 was challenge stress is positively related to employee performance, and results are (*β* = 0.16, *p* < 0.01), so H7 is also supported. After this, next hypothesis was challenge-related stress is positively related to employee creativity, and the results are (*β* = 0.18, *p* < 0.01), so H8 is also supported. Finally, following hypothesis 9 was creativity is positively related to employee performance, and the outcome is (*β* = 0.39, *p* < 0.01); hence, H9 also supported. Consequently, eight hypotheses are significantly endorsed according to the path analysis findings, and one (H1) is not supported (See Table 4).

**Table 4:** Results of hypothesis tests and model fitness index.

Hypotheses			Estimate	C.R.	*P*
EMPR	<−	OINO	0.06	1.463	0.143
EMPR	<−	TRAL	0.50	10.78	^***^
EMPR	<−	HSTR	−0.13	−3.59	^***^
EMPR	<−	CSTR	0.16	3.06	^***^
CRTV	<−	CSTR	0.18	4.16	^***^
CRTV	<−	HSTR	−0.08	−1.91	0.055
CRTV	<−	TRAL	0.37	8.09	^***^
CRTV	<−	OINO	0.48	8.75	^***^
EMPR	<−	CRTV	0.39	7.58	^***^

CMIN /df = 2.448, *p* ≤ 0.00; df = 270, GFI = 0.935, AGFI = 0.909, NFI = 0.934, CFI = 0.941, TLI = 0.935; RMSEA = 0.059, RMR = 0.091.

Here is the structural model described in Figure 1.

## Discussion and Conclusion

The study’s objective was to investigate the effect of organizational innovation, transformational leadership, and hindrance and challenge work stressor on employee performance among SMEs in Pakistan. Several kinds of research have been carried out separately on innovation and culture, but the current study provides a ground for the new dimensions in the innovation and culture concerning SMEs. Through this work, employers can identify how they can have a creative culture by using these factors. Every country in general and every developing country, in particular, seeks to strengthen and expand the business sector to promote economic growth and stability (Mahmood et al., 2019). SMEs are a key economic component of every country and can serve as a platform for the social and economic growth of the country. Toma et al. (2014) said SMEs play a critical element in any region or country’s economic growth. It added that SMEs are a key tool for people and governments by helping to reduce unemployment and achieve economic growth (Chauhan et al., 2019).

Our results showed that organizational innovation, transformational leadership, and challenge stressors positively affect employee performance and creativity, except organizational innovation has no impact on employee performance. One probable explanation is that training in many companies focuses on common knowledge or skills and job performance. Innovation, on the other hand, requires not only the ability to comprehend task-relevant approaches but also the ability to exceed logical and sequential thinking in order to make the leap to performance. That means, innovation is not the only dimension that enhances employee performance. There are other factors too that may need to develop the performance of the employees. Also, sometimes innovation comes up with change, and most people avoid change. So SMEs have to consider other variables too that may enhance employee performance, but organizational innovation is also important for the overall betterment of the business. This result is in line with previous findings of Hanifah et al. (2017) and Al-Amri et al. (2018). On the other hand, Hindrance stressors are considered to manipulate and threaten as opposed to challenge stressors. Our findings are similar to previous research that showed that hindrance stressors are negatively linked to an organization’s results (e.g., worker satisfaction, creativity, and performance; LePine et al., 2005; Podsakoff et al., 2007).

Finally, we found that creativity leads to the performance of the employees, so the significance of the finding demonstrates that employee creativity can help individuals increase their abilities, knowledge, and experience and thereby achieve organizational objectives. Using the creativity approach, workers will understand existing challenges and combine initiative and traditional concepts to generate new solutions to problems. Some previous studies agree with this finding (e.g., Hernández-Perlines et al., 2019). Employees reciprocate by putting more effort into their tasks, being more eager to contribute suggestions, and experimenting with new ways of doing their jobs when they believe the organization values them through sharing profits (incentive rewards) and giving them fascinating and important work. When people collaborate and watch their peers’ innovative habits, their performance improves. In summary, HRM practices can boost an organization’s total performance by impacting both the ability and motivation to be creative at the individual level. Previous research has found a link between total staff creativity and organizational performance (e.g., Paton and McCalman, 2008).

Employees who are innovative want to come up with new and valuable ideas. According to Nasir et al. (2020b), creative employees’ fresh ideas can be transferred to other employees within the company for their usage and development. As a result, individual innovation could contribute to the development of novel products at the organizational level. Overall, these findings imply that by offering quality leadership styles, innovation, and challenge stressor aspects, SMEs may improve their workforce’s overall creativity and performance. Employees with creative potential should be promoted, and reward systems and job design should be used to encourage employee motivation to be creative. Despite the literature revealing a favorable relationship between leadership styles and employee performance and creativity (Hsieh, 2021), there is a vacuum in knowledge about the relationship between leadership styles and employee performance. The current study revealed that employee creativity fully supports employee performance. The findings also imply that elements such as transformational leadership style, organizational innovation, and challenge stressors can all play a role in motivating employees to foster creativity in the workplace. In terms of empirical contribution, this study used firsthand data from SMEs from several companies, avoiding the investigation’s single-source bias. From a practical point of view, our research improves managers’ understanding of study variables in HRM functions such as recruiting and selection, reward, job design, teamwork in the hiring and promotion of employees, and performance measurement. Furthermore, our findings imply to practicing managers and the Pakistani government that a good firm’s environment practices might foster innovation. These insights may be helpful in making organizational and policy decisions.

### Future Implications and Limitations

Results of this study can be used to improve employee performance in the workplace by shaping plans for employee development. Business executives might learn more about how to engage and motivate staff to increase performance. The results can help corporate managers improve strategies and practices. The findings could be used by business leaders to identify issues which maintain employee engagement, progress performance, and boost revenue. Future study can employ the configuration approach to examine the connection between HRM practices and their impact on organizational creativity and employee performance. It may be required to conduct industry-specific research in order to employ objective indicators of innovation and performance. This study gives preliminary insights into the relationship between employee creativity, transformational leadership, work stressors, organizational innovation, and employee performance in a collection of SMEs in Pakistan. There are certain limitations to this research. For example, because survey questionnaires were employed to collect data for this study, it is strictly quantitative. Second, the conclusions are based on data from a single country, with a focus on SME employees. The results are limited to employees of SME sector. Future research could be conducted in foreign markets with a variety of sample sizes. Future research should use a longitudinal approach, with creativity and performance measured at least a year after study variables are measured. Also study can be performed in other areas of SMEs.

## Data Availability Statement

The raw data supporting the conclusions of this article will be made available by the authors, without undue reservation.

## Author Contributions

All authors listed have made a substantial, direct, and intellectual contribution to the work and approved it for publication.

## Conflict of Interest

The authors declare that the research was conducted in the absence of any commercial or financial relationships that could be construed as a potential conflict of interest.

## Publisher’s Note

All claims expressed in this article are solely those of the authors and do not necessarily represent those of their affiliated organizations, or those of the publisher, the editors and the reviewers. Any product that may be evaluated in this article, or claim that may be made by its manufacturer, is not guaranteed or endorsed by the publisher.
